# Single-Cell Sequencing Analysis and Weighted Co-Expression Network Analysis Based on Public Databases Identified That TNC Is a Novel Biomarker for Keloid

**DOI:** 10.3389/fimmu.2021.783907

**Published:** 2021-12-22

**Authors:** Jiaheng Xie, Liang Chen, Yuan Cao, Dan Wu, Wenwen Xiong, Kai Zhang, Jingping Shi, Ming Wang

**Affiliations:** ^1^Department of Burn and Plastic Surgery, The First Affiliated Hospital of Nanjing Medical University, Nanjing, China; ^2^Department of General Surgery, Fuyang Hospital Affiliated to Anhui Medical University, Fuyang, China; ^3^Fourth School of Clinical Medicine, Nanjing Medical University, Nanjing, China; ^4^Department of Rheumatology and Immunology, Nanjing Drum Tower Hospital, The Affiliated Hospital of Nanjing University Medical School, Nanjing, China; ^5^Department of Dermatology, The First Affiliated Hospital of Zhengzhou University, Zhengzhou, China; ^6^Pancreas Center, The First Affiliated Hospital with Nanjing Medical University, Nanjing, Jiangsu, China

**Keywords:** keloid, single-cell sequencing, weighted co-expression network analysis, differential expression analysis, Tenascin-c

## Abstract

**Background:**

The pathophysiology of keloid formation is not yet understood, so the identification of biomarkers for kelod can be one step towards designing new targeting therapies which will improve outcomes for patients with keloids or at risk of developing keloids.

**Methods:**

In this study, we performed single-cell RNA sequencing analysis, weighted co-expression network analysis, and differential expression analysis of keloids based on public databases. And 3 RNA sequencing data from keloid patients in our center were used for validation. Besides, we performed QRT-PCR on keloid tissue and adjacent normal tissues from 16 patients for further verification.

**Results:**

We identified the sensitive biomarker of keloid: Tenascin-C (TNC). Then, Pseudotime analysis found that the expression level of TNC decreased first, then stabilized and finally increased with fibroblast differentiation, suggesting that TNC may play an potential role in fibroblast differentiation. In addition, there were differences in the infiltration level of macrophages M0 between the TNC-high group and the TNC-low group. Macrophages M0 had a higher infiltration level in low TNC- group (P<0.05).

**Conclusion:**

Our results can provide a new idea for the diagnosis and treatment of keloid.

## Introduction

Wound healing plays an important role in maintaining skin barrier function (1). The final form of the wound is a scar (2). Normal scarring does not extend beyond the wound and gradually diminishes (2). Keloids are pathological healing of wounds and have many properties similar to tumors (3). Its clinical manifestation is scar hyperplasia beyond the wound, invasive growth (4). In addition, it does not gradually fade, often accompanied by pain and itching discomfort (4). The most common areas of keloids are the chest, back, and earlobes (5). There are many methods used to treat keloids which include surgery, chemotherapy, radiotherapy and stress therapy (6). But current treatment options are generally unsatisfactory. Therefore, exploring the pathogenesis of keloid is helpful to promote the development of new treatment regimens. Chronic inflammatory state is common in keloid, in which a variety of immune cells and cytokines are involved in the formation and development of keloid (1). Exploring changes in the immune microenvironment of keloid can not only deepen our understanding of the pathogenesis of keloid, but also promote the treatment of keloid patients to improve the prognosis. Now, advances in public databases have made it possible to explore the genetic mechanism and immune microenvironment of keloids in depth.

Single-cell sequencing analysis is a revolutionary research method (7), allowing for the clustering of the cells to study the differences in gene expression among the groups and the differences in cell development (8). Keloids are composed of a variety of cells which include fibroblasts, myofibroblasts, and mesenchymal stem cells (3). Therefore, single-cell sequencing analysis provides a method to accurately analyze the genetic characteristics of keloids at the single-cell level.

Weighted correlation network analysis (WGCNA) is a systems biology method used to describe gene association patterns among different samples (9). It can be used to identify highly covaried gene sets and to identify candidate biomarker genes or therapeutic targets based on the interconnectivity of the gene set and the association between the gene set and phenotype (10).

In this study, we used public databases for keloid single-cell sequencing analysis, WGCNA, and differential expression analysis to explore genetic changes in keloids to find potential targets. In addition, we used clinical RNA sequencing samples from our center to verify the results. Our results provide a new target for keloids and help to understand the pathogenesis of keloids from a genetic perspective.

## Methods

### Data Processing

We downloaded two keloid RNA chip data sets (GSE44270 and GSE145725) and one keloid single-cell RNA sequencing data set (GSE163973) from the GEO database. After standardization, samples without grouping information were excluded, and finally, 32 samples were obtained in GSE44270, 19 samples in GSE145725, and 6 paired tissue samples in GSE163973.

### Single-Cell Quality Control and Dimension Reduction Clustering

1. We selected cells with more than 300 expressed genes, less than 5500 expressed genes in total, less than 10 percent mitochondrial genes, and less than 3 percent red blood cell genes. A total of 20,629 cells were retained. Then 3000 hypervariable genes were selected for analysis, and then the number of principal components (PCs) was set to 15 to obtain cell cluster clusters, and then these clusters were displayed in the form of a “tSNE” diagram. We then annotated the resulting cluster. First, we found the top 10 significant markers (**Supplementary File 1**) in each cluster using the “FindAllmarkers” function. Then all the cells were annotated by the “SingleR” package. SingleR is an R package commonly used for automatic cell type annotation of single-cell RNA-seq (scRNA-seq) data. Spearman correlation was calculated between the expression profile of each cell and that of the reference sample. Second, we define the score for each label as a fixed quantile of the correlation distribution (0.8 by default). Finally, we repeat this for all the labels, and the label with the highest score serves as an annotation for the cell. In this study, we annotated cells using the SingleR package, and finally obtained the 12 clusters (**Supplementary File 2**).

### Pseudotime Analysis

2. After all the cells were annotated by the “SingleR” package, by using the “subset” function of Monocle R package, we first extracted all the objects of fibroblasts, and randomly selected 1500 cells through the “sample” function for subsequent pseudotime analysis. Then we used the method of “DDRTree” to reduce the dimension of cells, and then calculated the type of cell differentiation state through the function of “reduceDimension”. We used “FindMarkers” function to find genes that had significant differences among different states. The top 5 most significant markers of each State are shown in **Supplementary File 3**. Finally, we use the “plot_cell_trajectory” function to display the graph of cell differentiation trajectory.

### Gene Enrichment Analysis

We used the “GOplot” package to perform gene function and pathway enrichment analysis on the differential genes obtained from the single-cell analysis and preserved the gene set with | logFC | >1& P <0.05. First, the top 20 most relevant gene sets were presented in bar chart. Then we extracted the top 5 most related gene sets and presented them in the form of a circle graph.

### Weighted Co-Expression Network Analysis in GSE44270

We conducted WGCNA analysis on GSE44270, selected the top 50 genes with variance, and eliminated the outlier samples. The samples are grouped into different modules by selecting the best soft threshold. At the same time, we also identified conservative modules and eliminated modules with Zsummary.qual<2. Finally, the sample groups were connected, and the correlation analysis between different modules and sample types was carried out by the “Spearson” method.

### Differential Gene Analysis in GSE145275

We conducted a differential analysis of GSE145275. The limma package was used to analyze the differences between groups of keloids and normal tissues after standardization and presented as heat maps. Then we set the filter (| logFC | > 1 & p < 0.05) to get differentially expressed genes, and display them in volcanic graph. Finally, we took the intersection of the differential genes obtained by single-cell analysis, the module genes obtained by WGCNA, and the differential genes obtained by GSE145725.

### RNA Sequencing of 3 Pairs of Clinical Samples in Our Center

RNA-sequencing was performed on 3 keloid specimens as well as the patients’ normal skin (at least 1 cm from the keloid lesion). These 3 patients were enrolled between March 2021 and April 2021. All of them signed informed consent forms. And this study was approved by the Ethics Committee of the First Affiliated Hospital of Nanjing Medical University (No. 2021-SR-418). Keloid and the surrounding 1cm of normal tissue were resected by the same surgeon. TruSeq chain mRNA Library Prep Kit (Illumina) was used to generate the Library. Next-generation sequencing was performed on Illumina NovaSeq6000 (Illumina Inc., 100 cycles, single-read sequencing). Sample quality was assessed by FastQC. Illumina NovaSeq6000 analyzed RNA sequencing data for more global analysis of genomic abnormalities. Data is preprocessed using standard pipelines that contain quality control indicators, such as FastQC and MultiQC. Sequence alignment based on STAR-RNA sequencer and sequencing reads allocated to genome features *via* Featurecots and Vomo-Transformed.

### Expression Analysis of Potential Biomarkers in Keloid and Normal Tissue

First, expression analysis of potential biomarkers was performed in two datasets, GSE163973 and GSE44270. Subsequently, RNA-sequencing data from three patients at our center were used for validation. Student’s t test was used for statistical analysis.

### Immune Infiltration Analysis

We assessed the level of immune cell infiltration in all samples of GSE44270 and GSE145275 by using the analysis method of CIBERSORT and set the parameter “PERM” as 1000. Then we divided keloid patients into high-expression group and low-expression group with the median expression value of the potential biomarker as the node and used a violin diagram to show the distribution of immune cells in the high- and low-expression group.

### Gene Set Enrichment Analysis (GSEA)

First, we downloaded the gene set of “msigdb.v7.0.entrez.gmt” from GSEA’s official website. Then, after the differential genes were sorted down according to logFC, GSEA analysis was performed, and the result of P <0.05 was retained. Finally, the enrichment results were demonstrated using the function “Gseaplot2”.

### Quantitative Real-Time Polymerase Chain Reaction (qRT-PCR)

We next conducted qRT-PCR experiment on 16 keloid patients, from whom the keloid tissue and peri-keloid normal tissue were taken for mRNA quantification. These 16 patients were enrolled between June 2021 and October 2021. All of them signed informed consent forms. And this study was approved by the Ethics Committee of the First Affiliated Hospital of Nanjing Medical University (No. 2021-SR-418). Keloid and the surrounding 1cm of normal tissue were resected by the same surgeon. Total cellular RNAs were isolated from cells using Trizol Reagent (Invitrogen, Carlsbad, CA, USA) according to the manufacturer’s instructions. The reverse transcription was conducted using the reverse transcription kit provided by Takara (Otsu, Shiga, Japan). Real-time polymerase chain reaction (RT-PCR) was performed using a QuantiTect SYBR Green PCR Kit (Takara), and on a Applied Biosystems QuantStudio 1 (Thermo, Waltham, MA, USA). Relative quantification was determined using the -2ΔΔCt method. The relative expression of messenger RNA (mRNA) for each gene was normalized to the level of glyceraldehyde-3-phosphate dehydrogenase (GAPDH) mRNA. The specific primer sequences adopted in this experiment are: TNC: Forward: 5’- AGTAACGGTGGTGGATTCTGG-3’; Reverse:5ʹ-CTTCCGGTTCGGCTTCTGTA-3ʹ.GAPDH:Forward:5ʹ-GAACGGGAAGCTCACTGG-3ʹ;Reverse: 5ʹ- GCCTGCTTCACCACCTTCT - 3ʹ.

### Statistical Analysis

All the statistical analyses were carried out based on R software. Comparisons between keloid and normal groups were performed using the student’s t-test. The Chi-squared test and Fisher’s exact test were performed to compare the clinical variables. ROC analysis was generated to determine the discrimination value of TNC. GSEA was conducted with the clusterProfiler package. Unless otherwise specified, P<0.05 is considered significant.

## Results

**Figure 1:**
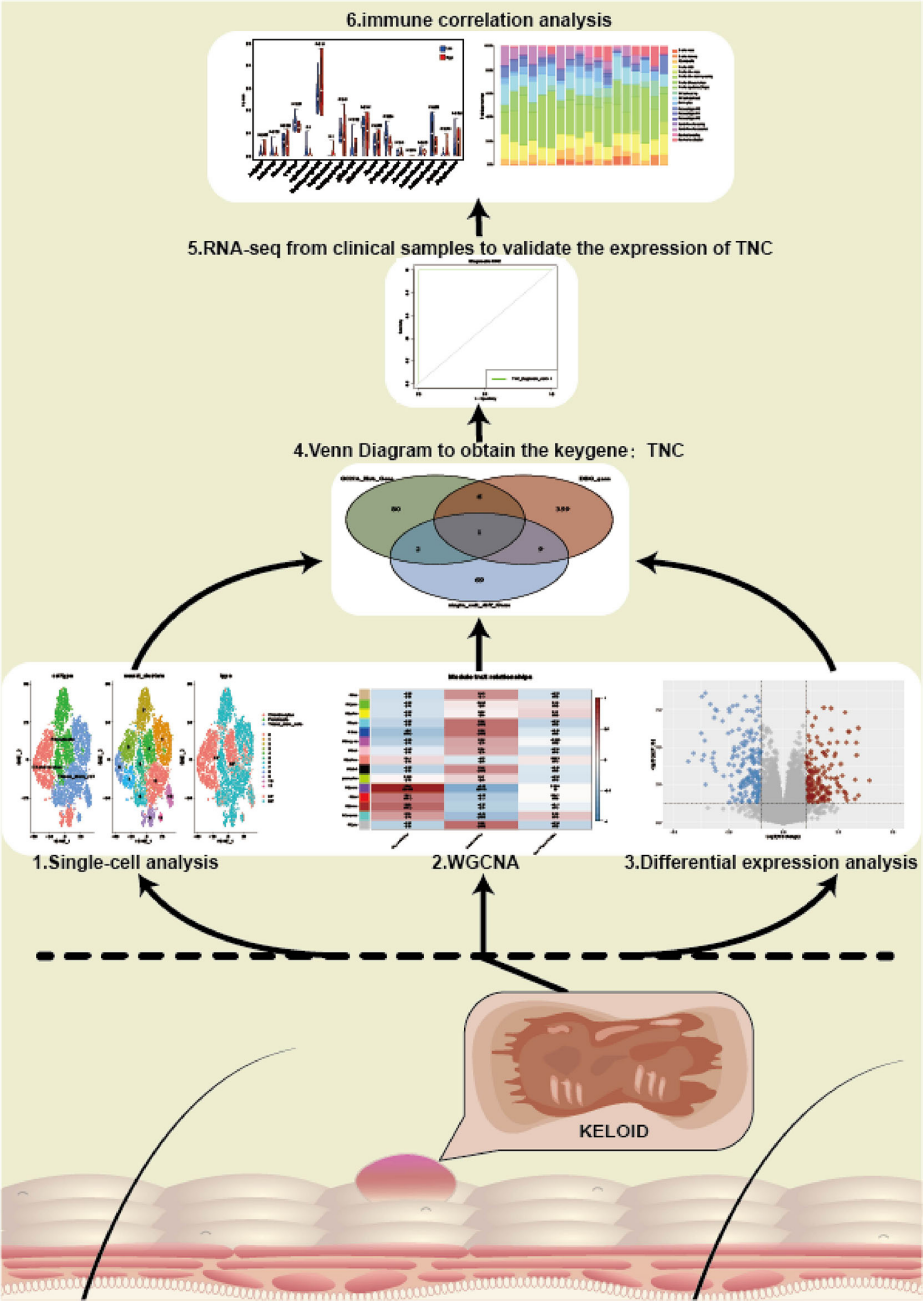
The flow chart of our study.

The flow chart of our study is shown in **Figure 1**.

### Single-Cell Quality Control and Dimension Reduction Clustering in GSE163973

**Figure 2:**
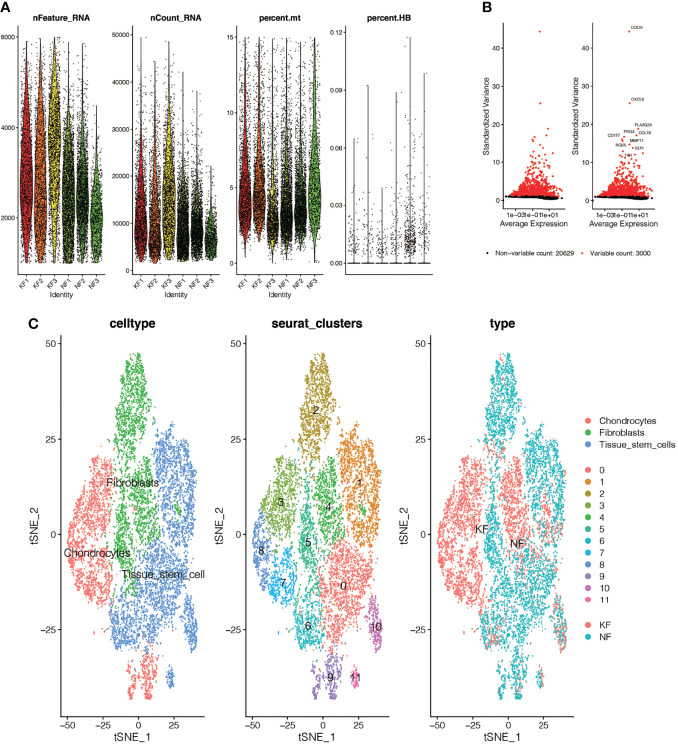
Single-cell quality control and dimension reduction clustering in GSE163973. **(A)** The percentage of mitochondrial genes and erythrocyte genes is limited to ensure the reliability of cell samples. **(B)** 3000 highly variable genes are shown in red, with the top 10 highlighted. **(C)** Dimensionality reduction and cluster analysis. The cells in the keloid dataset can be divided into 12 clusters, which can be roughly summarized as chondrocytes, fibroblasts and tissue stem cells.

We performed quality control on the single-cell data set. As shown in **Figure 2A**, we excluded some cells and limited the percentage of mitochondrial genes and red blood cell genes to ensure the reliability of cell samples. Then we picked out 3,000 highly variable genes and tagged the top 10. In **Figure 2B**, all the hypervariable genes are marked in red. In **Figure 2C**, we found that the cells were divided into 12 clusters, and different clusters were roughly summarized as chondrcocytes, fibroblasts, and tissue stem cells. In cluster3, 7, 8, and 9, which we annotated as chondrocytes, we found high expression levels of genes related to skeletal development, ossification, and osteoblasts, such as “COL11A1’,”COMP”, and “POSTN”. By reading the original text of this dataset, they probably should have been more accurately annotated as mesenchymal fibroblasts with high expression of osteogenic genes (8). Finally, we marked the keloid and normal samples as red and blue respectively.

### Pseudotime Analysis in GSE163973

**Figure 3:**
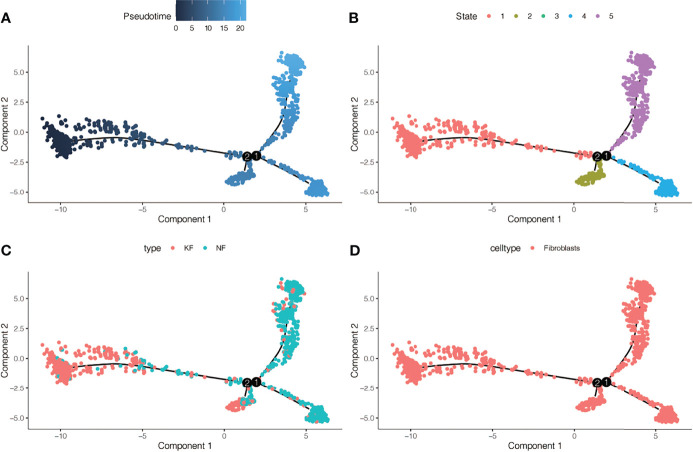
Pseudotime analysis in GSE163973. **(A)** Differences in the time sequence of cell differentiation. Darker blue indicates earlier differentiation, and lighter blue indicates later differentiation. This provides reference for subsequent analysis. **(B)** Five differentiation states of fibroblasts. The differentiation of State 1 is the earliest. **(C)** Differences in differentiation between keloid fibroblasts (KF) and normal fibroblasts (NF). **(D)** All cells analyzed were fibroblasts.

We conducted simulation analysis on the cell trajectory differentiation of all the fibroblasts, and finally found that as shown in **Figure 3A**, the darker the blue is, the earlier the cell differentiation is, indicating that with the differentiation of time, the fibroblasts differentiate from the left to the right, and the lightest blue is the latest differentiated cell. As shown in **Figure 3B**, in the process of differentiation of fibroblasts, there were altogether 5 differentiated states, which were marked with different colors, and the red one on the left was found to be the earliest differentiated type. We then investigated the differentiation process between keloid and normal fibroblasts and found that as shown in **Figure 3C**, keloid fibroblasts differentiated earlier, while normal fibroblasts differentiated later. **Figure 3D** indicates that all the cells analyzed are fibroblasts.

### Enrichment Analysis in the Single-Cell Dataset GSE163973

**Figure 4:**
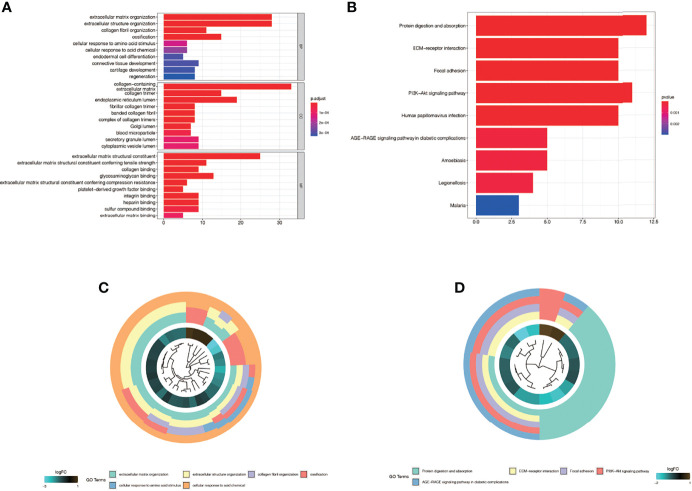
Enrichment analysis in the single-cell dataset GSE163973. **(A)** The Gene ontology (GO) enrichment. The results were divided into biological process (BP), cell component (CC) and biological function (MF). **(B)** Kyoto Encyclopedia of Genes and Genomes enrichment (KEGG). **(C)** The first five most significant GO enrichment results are shown in a circle graph. **(D)** The first five most significant KEGG enrichment results are shown in a circle graph.

We found differential genes between keloid fibroblasts and normal fibroblasts were enriched in many biological processes. **Figures 4A, C** show the results of GO enrichment analysis, which are divided into the biological process (BP), cell component (CC), and molecular function (MF). First of all, in BP, differential genes were mainly enriched in the extracellular matrix organization, extracellular structure organization, ossification and collagen fibril organization. In CC, differential genes were mainly enriched in collagen−containing, endoplasmic reticulum lumen, and other cell components. In MF, differential genes were mainly concentrated in extracellular matrix structural constituent, glycosaminoglycan binding, and extracellular matrix structural constituent conferring tensile strength and other functions. **Figures 4B, D** show the results of the KEGG enrichment analysis. Differential genes were mainly enriched in protein digestion and absorption, PI3K−Akt signaling pathway, focal adhesion, and ECM−receptor interaction.

### Weighted Co-Expression Network Analysis in GSE44270

**Figure 5:**
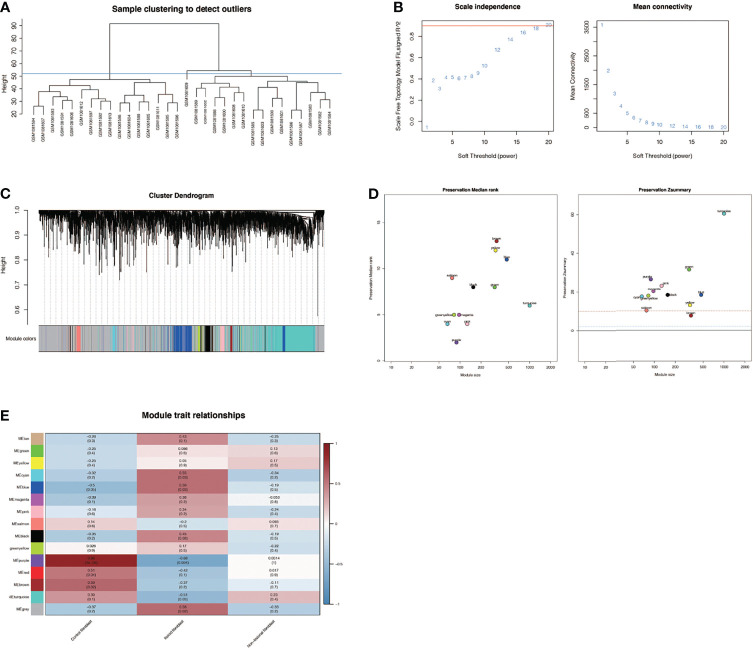
Weighted co-expression network analysis in GSE44270. **(A)** Sample clustering of dataset GSE44270. The samples were clustered into two significantly different clusters. The cluster on the left is selected for subsequent analysis. **(B)** Selection of optimal thresholds. The threshold is 18. **(C)** By aggregating genes with strong correlations into the same module, different modules are obtained and displayed in different colors. Turquoise modules account for a larger proportion. **(D)** Verification of the accuracy of WGCNA results. **(E)** Correlation analysis between modules and keloid. Purple module was significantly positively correlated with normal skin fibroblasts (NF) (COR= 0.86,P <0.001) and negatively correlated with keloid fibroblasts (KF) (COR = -0.68,P <0.01). The genes in the purple module were labeled as the WGCNA-hub genes.

By clustering 32 samples in the dataset, we found that the 16 samples on the left and the 16 samples on the right were quite different and clustered into 2 clusters (**Figure 5A**). After setting the cutoff value to 55, we included the left 16 samples in the subsequent analysis. We found that as the threshold increased, the R^2 value increased and crossed 0.8 (**Figure 5B**). We chose the optimal threshold of 18. Then, we clustered similar genes into different modules. The results showed that the turquoise module accounted for a larger proportion of the genes (**Figure 5C**). To verify the accuracy of the WGCNA process and results, the dataset was randomly divided into a training set and a testing set, and conservative sequences were identified and retained. Finally, the values of yellow, red, and tan modules were found to be lower than 2, which were excluded, and the final findings were shown in **Figure 5D**. Then, through correlation analysis between different modules and phenotype files, it was finally found that purple modules were significantly positively correlated with normal skin fibroblast (correlation = 0.86, P <0.001, **Figure 5E**), but significantly negatively correlated with keloid fibroblast (correlation = -0.68, P <0.01, **Figure 5E**). At the same time, there is no significant correlation in non−lesional fibroblast, suggesting that the purple module plays an important role in keloid fibers and normal fibers, which may be related to the occurrence and development of keloid. The genes in the purple module were labeled as the WGCNA-hub genes.

### Differential Gene Analysis in GSE145275

**Figure 6:**
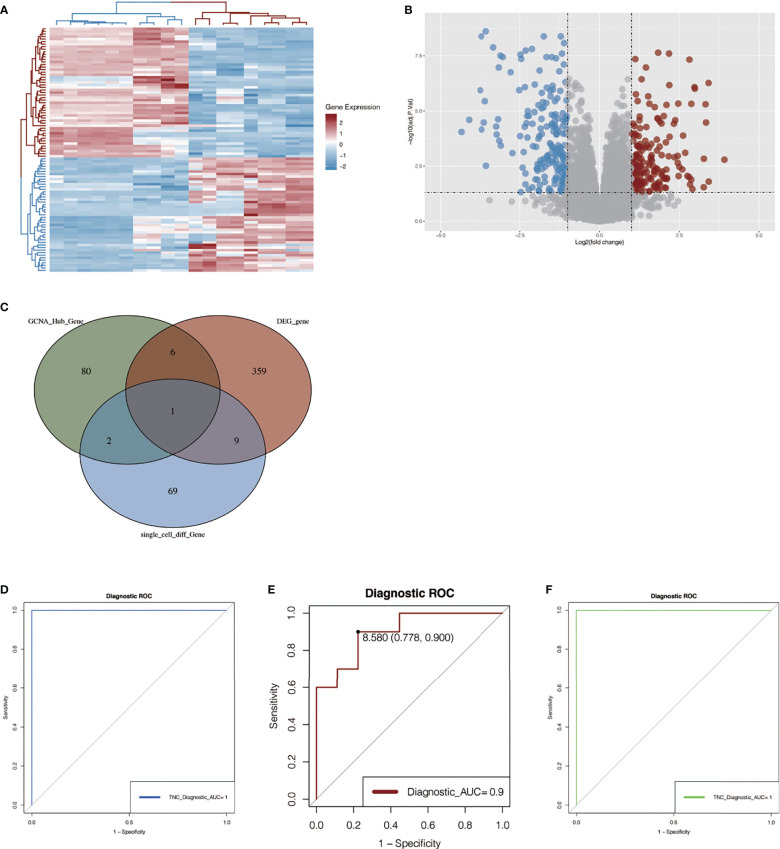
Differential gene analysis in GSE145275 and venn map intersection to obtain the key gene: TNC. **(A)** Heat maps of difference analysis results. The darker the red, the higher the expression level, and the darker the blue, the lower the expression level. **(B)** Volcanic maps of differential genes. Blue represents genes that are low-expressed in keloid tissue and red represents genes that are high-expressed in keloid tissue. **(C)** The intersection of single cell analysis, WGCNA and differential expression analysis was displayed in Venn diagram. The most significant gene, TNC, was obtained. **(D, E)** ROC curve construction in public data sets to evaluate the diagnostic accuracy of TNC of keloid. The AUC of dataset GSE145725 was 1.0 **(D)**. The AUC of ROC curve of dataset GSE44270 was 0.9 **(E)**. **(F)** RNA-sequencing data of our center were used to construct ROC curve to verify the accuracy of TNC. The AUC of the area under the curve is 1.

We performed differential expression analysis on another chip data set, GSE145275. As shown in **Figure 6A**, we found that the expression of these genes differed greatly among different samples, and the redder the color was, the higher the expression level was. In **Figure 6B**, differentially expressed genes are shown in the form of a volcano map, where blue represents the genes that are low in keloid tissue and red represents the genes that are high in keloid tissue.

### The Key Gene TNC Was Obtained by Venn Map Intersection

The differential genes obtained by single-cell analysis, the differential genes obtained by WGCNA analysis, and the differential genes in the dataset 145725 were collected by Venn graph (**Figure 6C**). Finally, a key gene TNC was obtained from the intersection, suggesting that this gene may play an important role in the development of keloid. Then, we plotted ROC curves in two chip data sets and found that the AUC of the ROC curve in data set GSE44270 was 0.9 (**Figure 6E**), and that in data set GSE145725, the AUC was 1.0 (**Figure 6D**). It is suggested that the TNC gene has a good effect on the diagnosis of keloid fibrous tissue and normal fibrous tissue. To further verify the above results, ROC curves were also drawn in the three pairs of keloid and normal samples sequenced by us, and the AUC value of the area under the curve was 1 (**Figure 6F**). Although the small sample size may affect the ROC value, it can be seen from the results that this gene also has a good effect on the diagnosis of keloid and normal tissue.

### Expression of TNC Gene in Keloid and Normal Tissue

**Figure 7:**
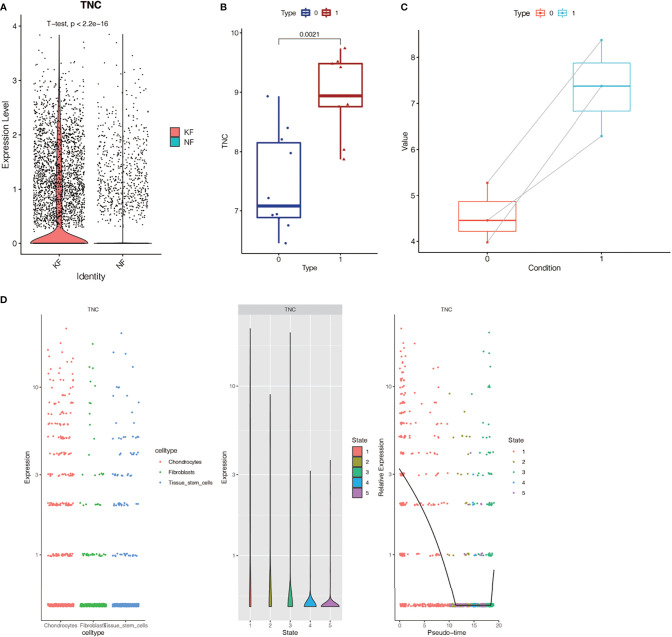
Expression of TNC gene in keloid and normal tissue. **(A)** Analysis of TNC expression in single cell sequencing dataset GSE163973. TNC expression in keloids was different from that in normal tissues (P < 2.2E-16). **(B)** TNC expression analysis in dataset GSE44270. Group 1 was keloid group, and group 0 was normal control group. TNC expression is up-regulated in keloid. **(C)** RNA-sequencing data in our center showed that TNC expression was up-regulated in keloid. **(D)** Correlation analysis between TNC and fibroblast differentiation. The expression of TNC gene decreased first and then increased with the differentiation of fibroblasts.

In the single-cell sequencing dataset, we found that the expression of TNC was different between keloid samples and normal samples (P < 2.2E-16, **Figure 7A**). Subsequently, we found that the expression of this gene was also different in keloid tissue and normal tissue in dataset GSE44270 (**Figure 7B**). Group 1 represented the keloid group and group 0 was the normal control. TNC expression is up-regulated in keloids. Then we used our RNA sequencing data to verify the expression of TNC in keloid and normal tissue and found that TNC was also up-regulated in keloids (**Figure 7C**). Raw RNA-sequencing data are summarized in **Supplementary File 4**. The pseudo-time series analysis mentioned above has found that fibroblasts in normal and keloid cells differ in cell differentiation trajectory, with a total of 5 differentiation states (**Figures 3B, C**). Next, we studied the significance of TNC in pseudo-time series analysis, as shown in **Figure 7D**. In the process of fibroblast differentiation, the expression of gene TNC first decreased, then tended to be stable, and finally increased again, suggesting that gene TNC may be related to fibroblast differentiation.

### Immune Infiltration Analysis

**Figure 8:**
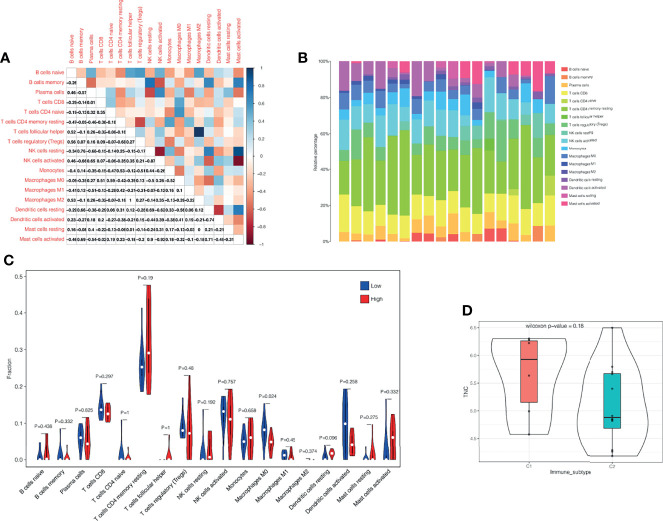
Immune infiltration analysis. **(A)** Correlation analysis of different immune cells. The darker the red, the stronger the negative correlation, and the darker the blue, the strongest positive correlation. **(B)** The immune landscape of keloid. The proportion of T cells in keloid immune microenvironment is the highest. **(C)** According to the expression of TNC, we divided them into TNC high expression group and TNC low expression group. The infiltration level of macrophage M0 was different between the two groups. **(D)** Correlation analysis of immune subtypes and TNC. TNC showed a high expression trend in the C1 immune subtype, but the difference was not statistically significant (P=0.18), which might be related to the small sample size.

We selected 18 samples of all keloid tissues from two RNA microarray datasets (GSE44270 and GSE145275) and performed immune infiltration analysis. **Figure 8B** shows the composition of immune cells in each sample. It was found that the dominant immune cells in keloid tissue were T cells. We then performed correlation analysis for these immune cells (**Figure 8A**), with the darker red indicating a stronger negative correlation and the darker blue indicating the strongest positive correlation. We found that there was a strong correlation between NK cell activated and NK cell resting, and a strong correlation between Mast cell activated and NK cell activated in keloid tissue. Mast cells activated cells and NK cells resting cells had a strong negative correlation, while Macrophage M2 and T cells follicular helper had the strongest negative correlation. Finally, according to the expression of TNC, we divided them into TNC high expression group and TNC low expression group. The infiltration level of macrophage M0 was different between the two groups (**Figure 8C**). Macrophages M0 had a higher infiltration level in low TNC- group (P<0.05). By using the “ImmuneSubtypeClassifier” R package, keloid patients were divided into different immune subtypes. By evaluating each sample, the best corresponding subtype was determined, and two subtypes were found, namely immune subtype C1 and immune subtype C2 (**Figure 8D**). Then, we explored the expression of TNC among immune subtypes and found that there was a trend of high expression of TNC in subtype C1, but P =0.18 indicated that the difference was not statistically significant, which may be caused by the small sample size (**Figure 8D**).

### GSEA Enrichment Analysis

**Figure 9:**
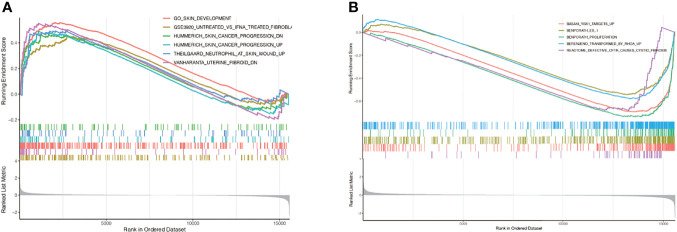
GSEA enrichment analysis. **(A)** In the group with high expression of TNC in keloids, the enrichment pathways included skin development, fibroblast, cancer progression, neutrophil at skin wound, and uterine fibroid; **(B)** In the low TNC expression group, the enriched pathways included YBX1 targets, ES-1, proliferation, transformed by RhoA, and defetive CFTR cause cystic fibrosis.

GSEA analysis found that, in the group with high expression of TNC in keloids, the enrichment pathways included skin development, fibroblast, cancer progression, neutrophil at skin wound, and uterine fibroid (**Figure 9A**); In the low TNC expression group, the enriched pathways included YBX1 targets, ES-1, proliferation, transformed by RhoA, and defetive CFTR cause cystic fibrosis (**Figure 9B**).

### Quantitative Real-Time Polymerase Chain Reaction (qRT-PCR)

**Figure 10:**
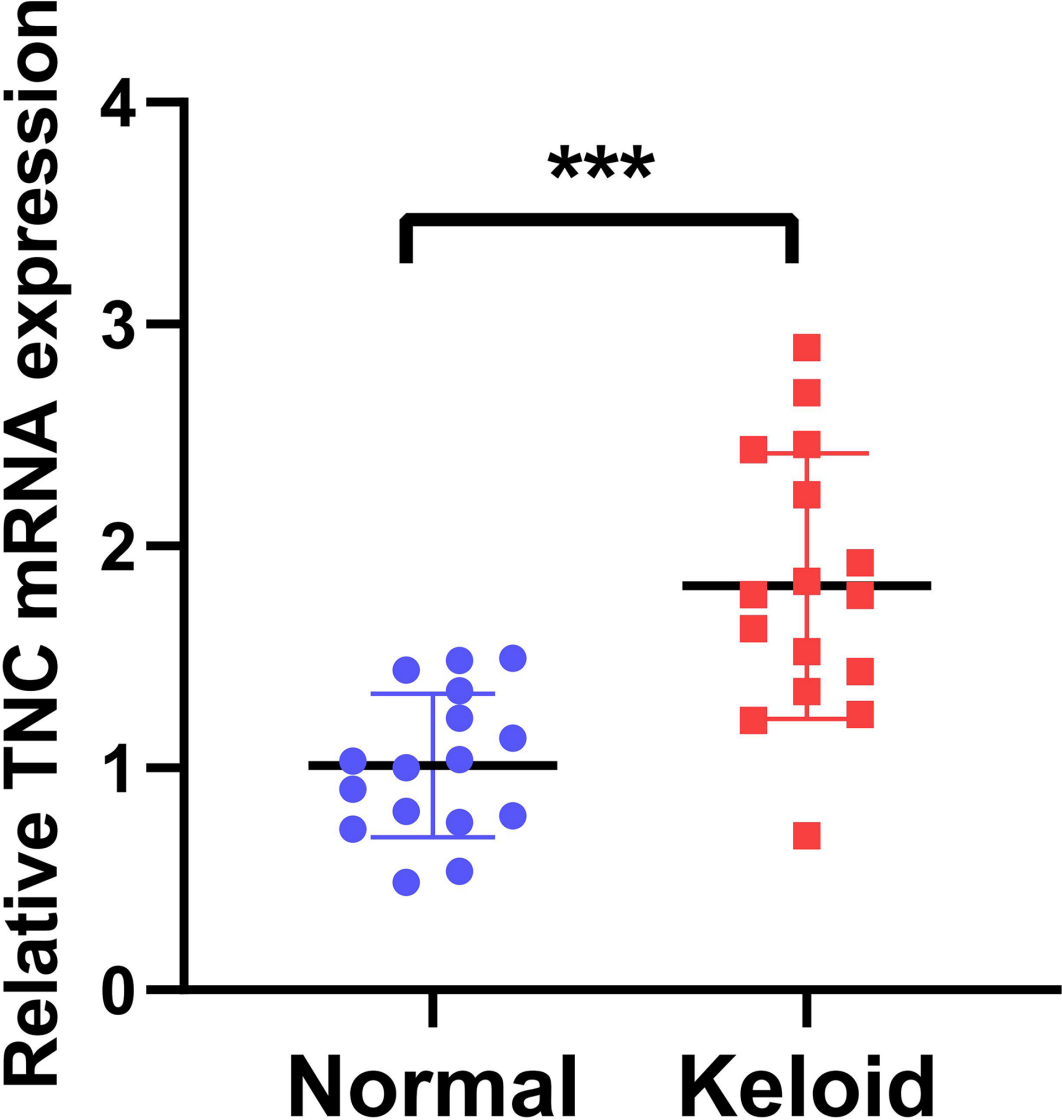
Quantitative Real-Time Polymerase Chain Reaction (qRT-PCR). The TNC expression in keloid was significantly higher than the Peri-keloid normal tissue from the same patient. (***p<0.001).

We next conducted qRT-PCR experiment on 16 keloid patients, from whom the keloid tissue and peri-keloid normal tissue were taken for TNC mRNA quantification. **Figure 10** showed that the TNC expression in keloid was significantly higher than the Peri-keloid normal tissue from the same patient. (***p<0.001).

## Discussion

Keloid is the abnormal proliferation of fibrous tissue in the skin of susceptible individuals after various traumatic or inflammatory stimuli (11). The hyperplasia of keloid often exceeds the range of original injury and is often accompanied by infection, which affects the aesthetic appearance and quality of life of many patients (12). Currently, conventional treatments for keloids do not work well. Hence, finding new biomarkers has significant significance for the treatment of keloid and the improvement of prognosis.

In this present study, through single-cell analysis, weighted co-expression network analysis, and differential expression analysis of the keloid data set, we finally found a gene TNC that was significantly associated with keloid. By constructing the ROC curve, we can see that TNC can accurately diagnose keloids. Then we validate the TNC expression in three clinical keloid samples from our center. Moreover, the up-regulation of TNC expression may be related to macrophage M0.

Tenascin-c (TNC) is the founding member of the Tenascin protein family (13). It was named because it was first discovered in a study of tendons (14). It binds to fibronectin and alters cell diffusion and signaling mediated by fibronectin and integrin (15). Therefore, TNC is widely regarded as a regulator of intercellular adhesion. Since the interaction of the extracellular matrix plays an important role in both homeostasis regulation and disease occurrence, TNC has extensive exploration value (16). TNC has been linked to several benign and malignant diseases, including heart disease, fibrosis, preeclampsia, rheumatoid arthritis, systemic lupus erythematosus, and cancer (17). In our study, after the intersection of WGCNA and single-cell sequencing analysis, only the TNC gene was obtained as a biomarker, indicating that TNC may play an important role in Keloid. Moreover, by constructing the ROC curve to calculate the AUC value, we can see that the AUC value is high, which indicates that TNC as a biomarker has high diagnostic accuracy. Thus, AUC is an accurate biomarker for Keloid.

Chronic inflammatory conditions in keloids are well known (18). Immune cells, immune-active substances, and signaling pathways are involved in the formation of the keloid immune microenvironment (19). These large members constitute the keloid immunomodulatory network and promote keloid growth (20). Macrophages have been shown to promote the transformation of fibroblasts into myofibroblasts by secreting transforming growth factor -β (TGF-β) and platelet-derived growth factor -CC (PDGF-CC), thereby promoting collagen deposition and keloid formation (21). Different subsets of T cells play different roles in the immune microenvironment (22). It is currently accepted by most people that interleukin-4 (IL-4) and IL-13 secreted by Th2 cells promote collagen synthesis and metabolism, leading to reticular fibrin deposition (23). Th1 cells inhibit the proliferation of fibroblasts by secreting IFN-γ, and down-regulate the expression of type I and III collagen genes, thereby reducing keloid formation (23). Tregs suppress other effector T cells and weaken the immune response (24). Mast cells, although common in allergic reactions, also play an important role in keloids (25). Mast cells stimulate fibroblast proliferation by releasing IL-4, VEGF, and basic fibroblast growth factor (bFGF), thereby increasing type I collagen synthesis (25). We can reduce keloid proliferation by blocking the PI-3K/Akt signaling pathway in mast cells (26). Neutrophils, as the first cells to arrive at the wound, also play a role in keloid formation (27). It can promote the formation of the immune microenvironment by recruiting monocytes and releasing immune-active substances. Our study provides an immune landscape of keloids, where we can see a larger proportion of T cells, suggesting that the role of T cells may be more significant. In addition, we also found that macrophage M0 was different between the high and low TNC expression groups. In the group with high TNC expression, the content of M0 in macrophages was lower, which provided a reference for further exploration of the immune microenvironment of keloid.

In summary, our study combined single-cell analysis, WGCNA, and differential expression analysis to deeply explore the genetic differences in keloids. The accuracy of the biomarker TNC we found is high, which can guide the diagnosis and treatment of keloid to a certain extent. Moreover, we verified the expression of TNC in clinical samples. However, our study also has deficiencies. We only used RNA-seq to verify the expression of TNC and the ROC curve, without relevant functional experiments to prove its function, which we will improve in the future.

## Conclusions

We identified a novel biomarker of Keloid, TNC, through single-cell analysis, WGCNA, and differential expression analysis. This biomarker was validated by RNA sequencing of our clinical keloid samples. In conclusion, our study provides a new reference for the diagnosis and treatment of keloid to some extent.

## Data Availability Statement

The datasets presented in this study can be found in online repositories. The names of the repository/repositories and accession number(s) can be found below: GEO, NCBI: GSE190626.

## Ethics Statement

The ethics committee has approved this study of the First Affiliated Hospital of Nanjing Medical University (No. 2021-SR-418). The patients/participants provided their written informed consent to participate in this study.

## Author Contributions

LC and JX designed the study. LC and DW were involved in database search and statistical analyses. LC, JX, and KZ were involved in the writing of manuscript and its critical revision. WX, YC, and MW was responsible for the submission of the final version of the paper. All authors approved the final version. All authors agree to be accountable for all aspects of the work.

## Funding

MW provided the funding. All authors declare that no conflict of interest exists. All the authors agreed on the final version of the manuscript.

## Conflict of Interest

The authors declare that the research was conducted in the absence of any commercial or financial relationships that could be construed as a potential conflict of interest.

## Publisher’s Note

All claims expressed in this article are solely those of the authors and do not necessarily represent those of their affiliated organizations, or those of the publisher, the editors and the reviewers. Any product that may be evaluated in this article, or claim that may be made by its manufacturer, is not guaranteed or endorsed by the publisher.
