# Supplementary figures and images for: Single-Cell Sequencing Analysis and Weighted Co-Expression Network Analysis Based on Public Databases Identified That TNC Is a Novel Biomarker for Keloid
Source: Front Immunol. 2021 Dec 22;12:783907. doi: 10.3389/fimmu.2021.783907 (PMC8728089; doi:10.3389/fimmu.2021.783907)

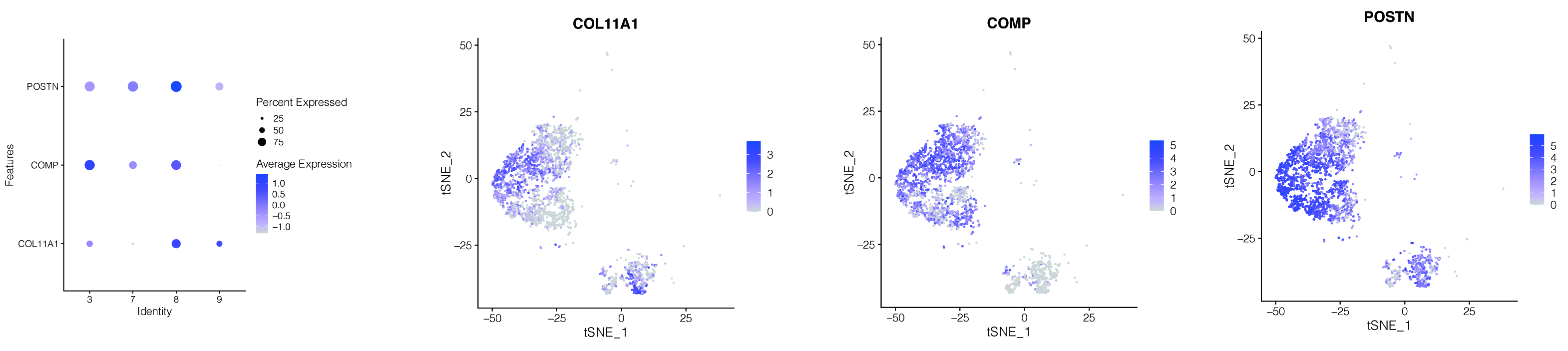

Supplement: Supplementary file 5 [file Image_1.tif]
